# Standardizing adverse drug event reporting data

**DOI:** 10.1186/2041-1480-5-36

**Published:** 2014-08-12

**Authors:** Liwei Wang, Guoqian Jiang, Dingcheng Li, Hongfang Liu

**Affiliations:** 1 Department of Medical Informatics, School of Public Health, Jilin University, Jilin, China; 2 Department of Health Sciences Research, Mayo Clinic, Rochester, MN, USA

## Abstract

**Background:**

The Adverse Event Reporting System (AERS) is an FDA database providing rich information on voluntary reports of adverse drug events (ADEs). Normalizing data in the AERS would improve the mining capacity of the AERS for drug safety signal detection and promote semantic interoperability between the AERS and other data sources. In this study, we normalize the AERS and build a publicly available normalized ADE data source. The drug information in the AERS is normalized to RxNorm, a standard terminology source for medication, using a natural language processing medication extraction tool, MedEx. Drug class information is then obtained from the National Drug File-Reference Terminology (NDF-RT) using a greedy algorithm. Adverse events are aggregated through mapping with the Preferred Term (PT) and System Organ Class (SOC) codes of Medical Dictionary for Regulatory Activities (MedDRA). The performance of MedEx-based annotation was evaluated and case studies were performed to demonstrate the usefulness of our approaches.

**Results:**

Our study yields an aggregated knowledge-enhanced AERS data mining set (AERS-DM). In total, the AERS-DM contains 37,029,228 Drug-ADE records. Seventy-one percent (10,221/14,490) of normalized drug concepts in the AERS were classified to 9 classes in NDF-RT. The number of unique pairs is 4,639,613 between RxNorm concepts and MedDRA Preferred Term (PT) codes and 205,725 between RxNorm concepts and SOC codes after ADE aggregation.

**Conclusions:**

We have built an open-source Drug-ADE knowledge resource with data being normalized and aggregated using standard biomedical ontologies. The data resource has the potential to assist the mining of ADE from AERS for the data mining research community.

## Introduction

Since the early 1990s, adverse drug events (ADEs) have received considerable attention from researchers in quality and patient safety [1]. Although randomized clinical trials (RCTs) are considered as a gold standard for determining the safety issues of drugs, it is generally recognized that premarketing RCTs may not detect all safety issues related to a particular drug in clinical practice [2]. The US Food and Drug Administration (FDA) Adverse Event Reporting System (AERS) is one of the main resources in post-marketed ADE detection based on data mining techniques [3,4]. The main data mining metrics used for ADE detection include the proportional reporting ratio (PRR), the reporting odds ratio (ROR), the information component (IC), and the empirical Bayes geometric mean (EBGM) [5]. For example, Kadoyama et al. [6] used the above metrics and detected signals for paclitaxel-associated mild, severe, and lethal hypersensitivity reactions and docetaxel-associated lethal reactions. Poluzzi et al. [7] detected drug-induced torsades de pointes (TdP) signals of linezolid, caspofungin, posaconazole, indinavir, and nelfinavir using ROR. However, most of existing studies on the AERS were carried out for a small number of drugs [6,8-10], and few studies were focused on large-scale mining or on detecting the etiology of ADE signals in terms of mechanism of action, physiologic effect, or molecular structure of drugs [11]. We realize that potential of the AERS has not been fully utilized, and one of main reasons for this is because there is a lack of standardization among drug names.

In the AERS, drugs can be registered by arbitrary names, including trade names, abbreviations, and even typographical errors since they are directly entered by health care professionals (e.g., physicians, pharmacists, nurses, etc.) and consumers (e.g., patients, family members, lawyers, etc.) [5]. There is limited normalization effort for the AERS data. For example, for drug names AERS uses “valid” trade names, if available, based on sources such as the Orange Book [12] and other internal databases [13]. Otherwise, the verbatim names are used, thus forming substantial barriers for data integration for the purpose of ADE signal detection. There have been some attempts in drug name normalization when mining AERS, but typically it is either unclear how the normalization was conducted or the normalization was attempted only for a small number of drugs [3,6,9-11,14-16].

In terms of ADE names, AERS does provide normalized terms based on Medical Dictionary for Regulatory Activities (MedDRA) preferred terms (PTs), though the use of MedDRA requires a license. In this study, we have demonstrated that MedDRA PT-based normalization actually enables the powerful data aggregation capability when we link the PT terms to their corresponding System Organ Class (SOC) categories.

In this study, we aim to produce an open-source AERS data mining set (AERS-DM), which is normalized and aggregated with two standard drug ontologies, including RxNorm and the National Drug File-Reference Terminology (NDF-RT), and one ADE terminology, MedDRA.

## Methods

### Resources

The FDA AERS database is a public database that includes 7 tables. Its structure is in compliance with the international safety reporting guidance (ICH E2B) [17]. Information related to a single AERS report can be retrieved from those tables using a unique identifier (i.e., Individual Safety Report (ISR) number). Among them, the DRUG table includes drug-related information such as “DRUG_SEQ” (a unique number for identifying a drug in a report), “DRUGNAME,” “ROUTE” (the route of drug administration), and “DOSE_VBM” (verbatim text for dose, frequency, and route, exactly as entered on the report). The REAC table includes adverse event information using PTs in MedDRA, a medical terminology adopted to describe adverse drug event [18]. The DEMO table includes patient demographics and administrative information of the events, including “CASE” (case number for identifying an AERS case) and “FDA_DT” (date FDA received report).

**Figure 1:**
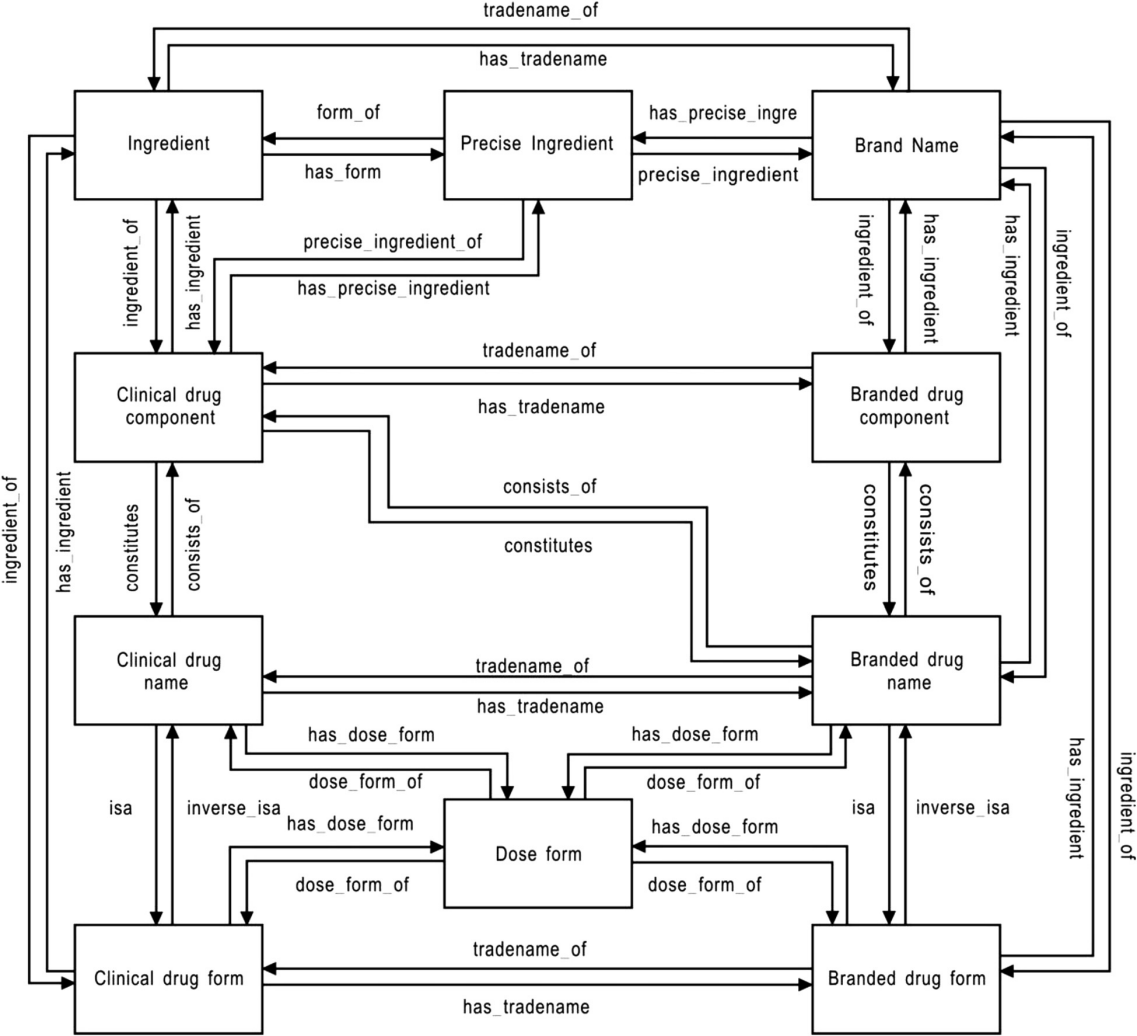
**Relations among RxNorm concepts, adapted from Peters and Bodenreider [**[21]**].**

RxNorm, released initially in 2004, is a standardized nomenclature for clinical drugs and drug delivery devices [19]. Since its creation, RxNorm has been increasingly recognized by the biomedical informatics community as an emerging standard for clinical information exchange [20]. RxNorm is organized by concepts, in which each concept consists of drug names sharing the same meaning at a specific level of abstraction and is assigned a concept unique identifier (RxCUI). RxNorm also provides relationships between concepts, as indicated in Figure 1, adapted from Peters and Bodenreider [21]. For example, 'Diphenhydramine Hydrochloride’ is the precise_ingredient_of ‘Tylenol PM’. The description of all such relationships can be retrieved, see the Availability and requirements section for the webpage. Data in RxNorm is distributed in Rich Release Format (RRF) tables, which is the default relational format used by the National Library of Medicine (NLM).

**Figure 2:**
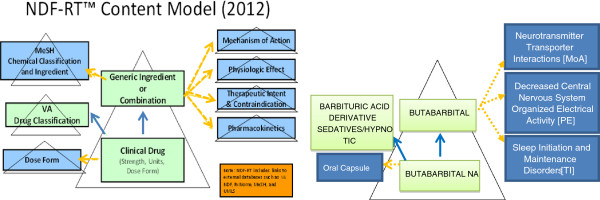
**NDF-RT content model and the example.** Triangles denote hierarchies of related concepts, categorized in the rectangles within the triangles. Taxonomic or ISA relationships (upward-pointing blue solid arrows) unify NDF-RT™ clinical drug concepts into a polyhierarchy, classified both by their VA drug class and their generic ingredient(s). Various named role relationships (sideways-pointing amber dash arrows) define the central drug concepts (green) from which they originate in terms of the reference hierarchy concepts (blue) pointed to. Role relationships are also inherited into subsumed clinical drug concepts. Adapted from NDF-RT document [April 2012 Version] [23].

NDF-RT was developed by the Veterans Health Administration, providing clinical information about medications, and has been included in RxNorm. NDF-RT uses a description logic-based, formal reference model that groups drugs and ingredients into the high-level classes for Chemical Structure (e.g., *Acetanilides*), Mechanism of Action (e.g., *Prostaglandin Receptor Antagonists*), Physiological Effect (e.g., *Decreased Prostaglandin Production*), drug-disease relationship describing the Therapeutic Intent (e.g., *Pain*), Pharmacokinetics describing the mechanisms of absorption and distribution of an administered drug within a body (e.g., *Hepatic Metabolism*), and legacy VA-NDF classes for Pharmaceutical Preparations (VA Drug Class; e.g., *Non-Opioid Analgesic*) [22]. Figure 2 shows the NDF-RT content model [23] together with an example showing drug class information for BUTABARBTIAL NA 100MG CAP.

We utilized RxNorm and NDF-RT for normalizing and aggregating drug information in AERS in consideration of three reasons. First, these two ontologies are publicly available medication ontologies that have been intensively developed and used for drug data integration [22,24,25]. Second, RxNorm aims to enable various systems using different standardized drug nomenclatures to share and exchange data efficiently, which we believe meets the requirements for meaningful use of the ADE reporting data. In addition, since RxNorm only represents a nomenclature of drugs and does not contain drug categorical information, we leveraged the categorical information extracted from NDF-RT for medication data aggregation. Third, as a part of the Unified Medical Language System (UMLS), RxNorm and NDF-RT can function as interoperable drug standards that can integrate with other health data, such as electronic health records (EHRs), so as to facilitate the semantic integration of the data in the health domain.

Finally MedDRA is a controlled terminology developed for reporting adverse events, related to drugs, to regulatory agencies [26]. MedDRA has a hierarchical structure with five levels: SOC, High-Level Group Term (HGLT), High-Level Term (HLT), PT and Lowest-Level Term (LLT). There are 26 classes (SOCs). PTs are the main descriptors in MedDRA and are used in AERS. All MedDRA terms are integrated UMLS.

### Tool

In our study, we used a natural language processing (NLP) tool, MedEx, to normalize AERS drugs. MedEx extracts medication information from clinical notes [27]. Besides MedEx, there are a number of other existing NLP-based tools available that could be used for drug normalization, including MedLEE [28], National Center for Biomedical Ontology (NCBO) Annotator Web Service [29], and Mayo cTAKES [30]. We chose to use the MedEx because we consider it an optimized system for drug normalization with a relative good performance, ranked second in the 2009 i2b2 Medication Extraction challenge, where the first-place system in that challenge is not available for public use [31]. The evaluation showed that MedEx performed well on identifying drug names, with precision (97%), recall (88%) and F-measure (92%) for 50 discharge summaries and precision (95%), recall (92%) and F-measure (93%) for 25 clinic notes, respectively [27]. In this study, we used MedEx version 2.0. Input files for MedEx included concrete information on drugs, and output normalized data included RxNorm codes.

### Data processing

**Figure 3:**
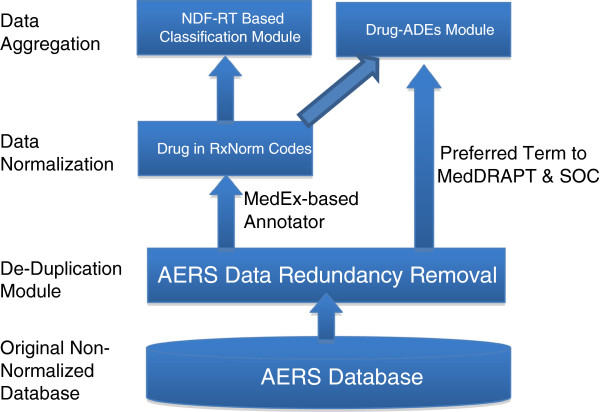
An overview of the data processing flow.

Figure 3 presents an overview of the data processing flow that contains three steps: de-duplication, drug normalization, and data aggregation. In the de-duplication step, redundant reports are removed. In the normalization step, MedEx is applied to normalize AERS drugs to RxNorm codes. During aggregation, adverse events are aggregated according to MedDRA SOC and PT codes, and NDF-RT–based classification information for those drugs is obtained from RxNorm. Two tables are formed; one stores the normalized Drug-ADE information and the other stores the aggregated information of Drug-ADE. The data in the two tables can be connected through the RxNorm codes. Those steps are further detailed below.

### Removing redundant AERS data

According to the FDA’s recommended method for de-duplication, for reports with the same CASE number, we select the latest (most recent) report date (i.e., FDA_DT) in the AERS “DEMO” table. For reports with the same CASE and FDA_DT values, we select the one with the highest ISR number. Table 1 shows examples of how to delete duplicate reports. We select the ISR “4275741” for CASE number “4047837,” and ISR “7637797” for CASE number “8468457.” Consequently, the selected ISRs in the DEMO table are kept in DRUG and REAC tables.

**Table 1:** Examples of duplicate reports

**ISR**	**CASE**	**FDA_DT**	**De-duplication**
4269368	4047837	20040113	×
4275741	4047837	20040121	√
7637789	8468457	20110720	×
7637797	8468457	20110720	√

“ISR” is the unique number for identifying an AERS report. “CASE” is the number for identifying an AERS case. "FDA_DT” is the date FDA received report. “De-duplication” indicates the positive or negative action.

### Normalizing AERS drug and ADE data

After de-duplication, we concatenate the following fields in the DRUG table: “DRUGNAME,” “ROUTE,” and “DOSE_VBM, and the resulting strings are normalized with MedEx. Compared to DRUGNAME alone, the concatenated string gives more comprehensive information about the corresponding drug, since the “ROUTE” field can provide information such as “Oral” and the “DOSE_VBM” field can provide information such as “Tablet.” The results are mapped to the RxNorm code RxCUI. For example, the concatenated string “POTASSIUM CHLORIDE EXTENDED RELEASE TABLET EXTENDED RELEASE TABLET ORAL 20 MEQ BID ORAL” is normalized to RxCUI 198116 (i.e., “Potassium Chloride 20 MEQ Extended Release Tablet”). Meanwhile, we map “PT” entries of the REAC table to PT and SOC codes of MedDRA.

### Aggregating normalized drug and ADE data

**Figure 4:**
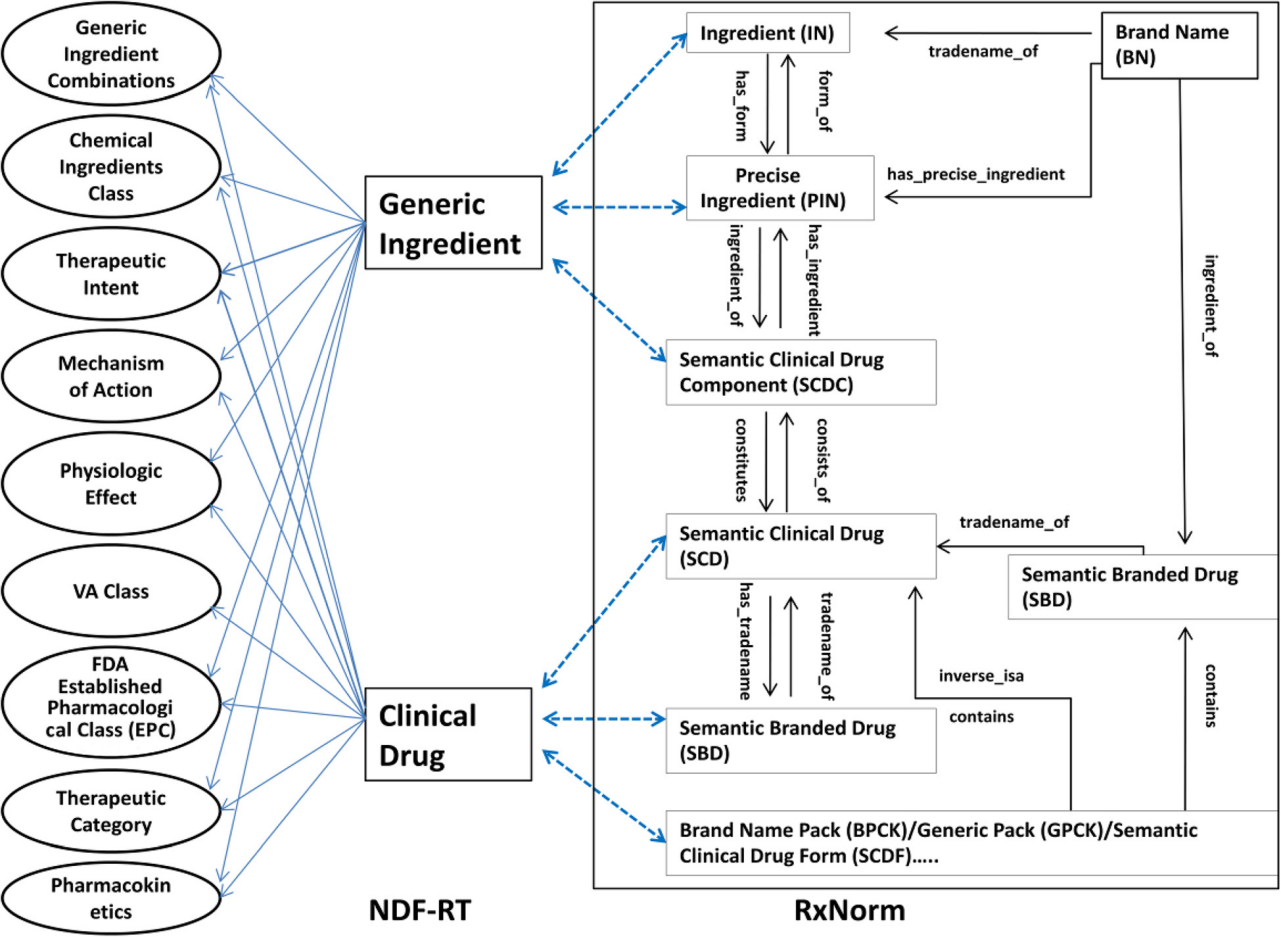
Traversal pathways for classifying AERS drugs based on NDF-RT.

The algorithm for classifying AERS drugs based on NDF-RT includes two parts; the first part is to identify the corresponding NDF-RT concepts for those normalized AERS drugs and the second is to extract the associated NDF-RT classification information.Specifically, RxNorm contains NDF-RT ingredients and clinical drugs. Meanwhile, NDF-RT ingredients are connected to their mechanisms of action, physiologic effects, and therapeutics (indications and contraindications) and the corresponding clinical drugs inherit those relations. NDF-RT clinical drugs are also connected to their corresponding VA drug classes. Therefore, if a given RxCUI itself is an NDF-RT concept (i.e., one of its sources is NDF-RT), we use it to find NDF-RT classification information. Otherwise, we traverse the relations provided by RxNorm to greedily identify the related NDF-RT ingredients and clinical drugs, and then extract the associated classification information (see Figure 4).

We obtain PT and SOC codes in MedDRA for “PT” entries in the AERS. For example, “PT” entries “Anaemia of chronic disease,” “Anaemia of malignant disease,” and “Nephrogenic anaemia” are mapped to corresponding PT codes “10002073,” “10049105,” and “10058116.” And these codes are aggregated under the SOC code “10005329” (i.e., “blood and lymphatic system disorders”).

#### Experiments

For the experiments, we first gathered the AERS data that are publicly available from 2004 to 2011 [32]. We used the May 2012 release of RxNorm (which contains the mappings to NDF-RT) and the 14.1 version of MedDRA. We ultimately produced an AERS-DM that is composed of two tables, one containing the normalized Drug-ADE information and the other containing the aggregation information of the Drug-ADEs. We then analyzed the statistics of the normalization and aggregation for AERS data in the AERS-DM. We also performed case studies to demonstrate the usefulness of the AERS-DM.

We evaluated the normalization performance of MedEx in two steps. For the first step, we randomly selected 200 unique input drug names before MedEx-based annotations. We recruited three reviewers with medical background who manually reviewed the 200 drug names and annotated them using the RxNorm codes. The version of the RxNorm used in the evaluation was the same as that included in MedEx. A gold standard was generated after the reviewers achieved inter-agreements. Precision (P), Recall (R), and F-measure (F) were calculated for these selected drug names, using P = TP/(TP + FP), R = TP/(TP + FN), and F = 2PR/(P + R), in which TP stands for True Positive, FP stands for False Positive, and FN stands for False Negative. For the second step, we randomly selected 100 drug names that failed to be normalized. Then we confirmed whether or not the drug names are included in RxNorm. In addition, we checked to see if an unmatched drug name is a valid domestic or foreign drug by using two drug resources. The first resource is Drugs@FDA, a drug dictionary providing FDA-approved brand and generic drug information [13], and the second resource is Drugs.com, the largest independent drug information website available on the Internet [33].

Additionally, we evaluated the validity of the algorithm used for identifying the mappings between RxNorm codes and NDF-RT concepts. We randomly selected 20 AERS drug names and manually checked the accuracy of the mappings produced by our algorithms.

## Results

### General statistics of normalization and aggregation

After de-duplicating reports, according to the recommended method in the download files provided by FDA [34], the number of AERS records is reduced to 2,643,979 from the original 3,874,965. The number of unique “verbatim” drug names is reduced to 1,517,811 from the original 1,700,925.

For drug name normalization, 1,125,045 of 1,517,811 (74%) AERS unique drug names were normalized to 14,489 unique RxNorm concepts, of which 10,221 (71%) were classified in NDF-RT.

**Figure 5:**
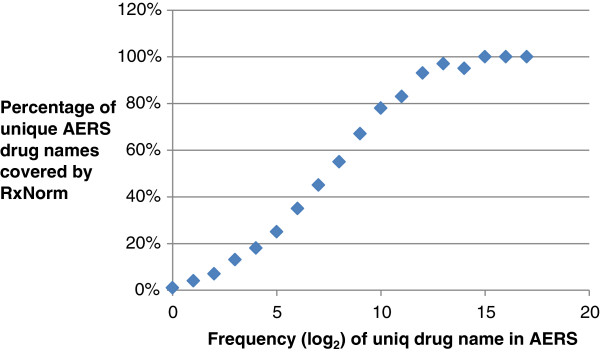
Distribution of unique drug names in AERS.

For the ADE normalization, we mapped 14,740 existing MedDRA PT terms in the AERS to MedDRA codes, accounting for 76% of 19,294 total MedDRA PT terms. These MedDRA PT codes were then mapped to 26 MedDRA SOCs.Figure 5 shows the distribution of unique drug names in the AERS (normalized with the single field “DRUGNAME”), which follows Zifp’s Law. In other words, the more popular the drug names are, the higher the chance those drug names are to be normalized by the RxNorm codes.

We classified the RxNorm concepts using the NDF-RT. Table 2 shows the coverage of AERS drugs by NDF-RT classes. For example, 5,823 RxNorm concepts were mapped to 29 corresponding VA classes, accounting for 57% of all classified RxNorm concepts and corresponding to 77% of total AERS reports. Table 3 shows the results of MedDRA PT code aggregation by MedDRA SOCs.

**Table 2:** Coverage of AERS drugs by NDF-RT classes

**Drug_class_type**^**1**^	**Drug_class, No.**^**2**^	**RxNormConcept, No. in the AERS**^**3**^	**a%**^**4**^	**b%**^**5**^
Generic Ingredient Combinations	26	9,813	96	88
Chemical Ingredients Class	16	9,331	91	81
Therapeutic Intent	24	8,069	79	78
Mechanism of Action	7	8,061	79	81
Physiologic Effect	16	7,989	78	82
VA Class	29	5,823	57	77
FDA Established Pharmacologic Class (EPC)	66	3,049	30	46
Therapeutic Category	7	2,880	28	50
Pharmacokinetics	1	730	7	28
Total	192	10,221	100	100

^1^“Drug_class_type” represents various classes provided by NDF-RT as shown in Figure 2.

^2^“Drug_Class, No.” is the number of direct subordinate classes of each Drug_class_type.

^3^“RxNorm concept, No. in the AERS” shows the total number of RxNorm concepts in the AERS for each drug class type.

^4^“a%” indicates the percentage of classified RxNorm concepts for each Drug_class_type, calculated as the division of “RxNorm concept, No. in the AERS” by 10,221; a total of RxNorm concepts mapped to at least one NDF-RT class.

^5^“b%” indicates the percentage of classified AERS reports, calculated as the division of the number of AERS reports assigned with the Drug_class_type by the total AERS report number (2,643,979).

**Table 3:** Aggregation of MedDRA PT codes by MedDRA SOCs

**MedDRA SOC Code**	**MedDRA SOC Terms**	**PT Codes, No.**^**1**^	**AERS Reports, No. (%)**^**2**^
10018065	General disorders and administration site conditions	595	875,070 (15.6)
10029205	Nervous system disorders	729	570,448 (10.2)
10017947	Gastrointestinal disorders	699	447,243 (8.0)
10022891	Investigations	2,748	407,270 (7.3)
10037175	Psychiatric disorders	453	329,198 (5.9)
10022117	Injury, poisoning, and procedural complications	697	311,952 (5.5)
10038738	Respiratory, thoracic, and mediastinal disorders	437	287,425 (5.1)
10028395	Musculoskeletal and connective tissue disorders	365	276,784 (4.9)
10040785	Skin and subcutaneous tissue disorders	390	273,674 (4.9)
10021881	Infections and infestations	1,361	267,741 (4.8)
	Infections and infestations		
10007541	Cardiac disorders	283	237,037 (4.2)
10047065	Vascular disorders	595	195,591 (3.5)
10027433	Metabolism and nutrition disorders	236	160,134 (2.9)
10038359	Renal and urinary disorders	288	132,055 (2.4)
10029104	Neoplasms benign, malignant and unspecified (incl. cysts and polyps)	1,293	129,227 (2.3)
10005329	Blood and lymphatic system disorders	218	122,276 (2.2)
10015919	Eye disorders	462	114,312 (2.0)
10042613	Surgical and medical procedures	1,176	89,005 (1.6)
10019805	Hepatobiliary disorders	162	79,067 (1.4)
10038604	Reproductive system and breast disorders	381	73,431 (1.3)
10021428	Immune system disorders	111	64,127 (1.1)
10041244	Social circumstances	189	48,077 (0.9)
10036585	Pregnancy, puerperium and perinatal conditions	182	36,959 (0.7)
10013993	Ear and labyrinth disorders	75	30,174 (0.5)
10010331	Congenital, familial, and genetic disorders	833	21,244 (0.4)
10014698	Endocrine disorders	129	18,053 (0.3)

^1^“PT Codes, No.” shows the number of PT codes for each SOC.

^2^“AERS Reports, No. (%)” shows the number of AERS reports and the percentage among the overall AERS report number for each SOC, where one report may contain several SOCs associated with several drugs.

### Statistics of AERS-DM

The AERS-DM includes two tables, as discussed above. There are 37,029,228 Drug-ADE records after de-duplication. The number of unique pairs between RxNorm concepts and MedDRA codes is 4,639,613, and between RxNorm concepts and SOC pairs after ADE aggregation, 205,725. Tables 4 and 5 show the top 10 most frequent pairs (Therapeutic Category, PT and Therapeutic Category, SOC), respectively.

**Table 4:** Therapeutic category and PT pairs

**Co-occurrence, No.**	**Therapeutic Category, Codes (Terms)**	**PT, Codes (Terms)**
31,858	2225 (Central Nervous System Agent)	10028813 (Nausea)
30,824	2225 (Central Nervous System Agent)	10013709 (Drug ineffective)
24,922	882 (Antirheumatic Agent)	10022086 (Injection site pain)
23,633	882 (Antirheumatic Agent)	10028596(Myocardial infarction)
20,240	2095 (Cardiovascular Agent)	10028813 (Nausea)
18,435	2095 (Cardiovascular Agent)	10013968 (Dyspnoea)
15,695	4703 (Gastrointestinal Agent)	10028813 (Nausea)
11,709	4703 (Gastrointestinal Agent)	10012735 (Diarrhoea)
10,509	884 (Anti-infective Agent)	10028813(Nausea)
10,505	884 (Anti-infective Agent)	10037660 (Pyrexia)
10,022	988 (Antineoplastic Agent)	10012735 (Diarrhoea)
8,865	988 (Antineoplastic Agent)	10011906 (Death)
100	106571 (Diagnostic Agent)	10022061(Injection site erythema )
69	106571 (Diagnostic Agent)	10028813(Nausea)

**Table 5:** Therapeutic category and SOC pairs

**Co-occurrence, No.**	**Therapeutic Category, Codes (Terms)**	**SOC, Codes (Terms)**
133,936	2095 (Cardiovascular Agent)	10018065 (General disorders and administration site conditions)
90,959	2095 (Cardiovascular Agent)	10029205 (Nervous system disorders)
194,311	2225 (Central Nervous System Agent)	10018065 (General disorders and administration site conditions)
175,172	2225 (Central Nervous System Agent)	10029205 (Nervous system disorders)
129,953	882 (Antirheumatic Agent)	10018065 (General disorders and administration site conditions)
61,386	882 (Antirheumatic Agent)	10029205 (Nervous system disorders)
76,007	4703 (Gastrointestinal Agent)	10018065 (General disorders and administration site conditions)
69,825	4703 (Gastrointestinal Agent)	10017947 (Gastrointestinal disorders)
65,105	884 (Anti-infective agent)	10018065 (General disorders and administration site conditions)
41,900	884 (Anti-infective agent)	10029205 (Nervous system disorders)
53,478	988 (Antineoplastic Agent)	10018065 (General disorders and administration site conditions)
35,625	988 (Antineoplastic Agent)	10017947 (Gastrointestinal disorders)
403	106571 (Diagnostic Agent)	10018065 (General disorders and administration site conditions)
251	106571 (Diagnostic Agent)	10040785 (Skin and subcutaneous tissue disorders))

### Evaluation results

The initial inter-annotator agreement on the 200 annotated drug names was 62.8%, a low agreement rate, maybe due to the various understandings in the rules for drug name normalization from the annotation training. It indeed indicated the difficulty of the drug normalization in AERS. Comparing MedEx-based annotations with the gold standard generated from majority votes by three human reviewers, we calculated the performance measures. TP, FP and FN were calculated as 138, 7 and 6 respectively. Recall, Precision and F-measure were then calculated as 95.8%, 95.2% and 95.5% respectively, which is comparable with performance measures in the original evaluation of MedEx [27]. The slight difference may be caused by different evaluation contexts, and different drug name numbers in gold standards, with discharge summaries (377), clinic notes (200) and AERS (200). The false positive was low in the 200 annotated drug names, which was mainly due to the false recognition of partial drug names, for example, both drug names “ADONA (CARBAZOCROME SODIUM SULFONATE)” and “CLEXANE (HEPARNI-FRACTION SODIUM SALT)” were normalized to “sodium (RxCUI 9853)”. The first, a foreign drug from Italy and Japan, is out of the scope of RxNorm, the second is covered by RxNorm but not identified. As a result both are falsely mapped by MedEx.

Table 6 shows the evaluation results of 100 drug names failed to be normalized by MedEx. A large portion of them (75) are due to problems associated with AERS records, including names not covered by RxNorm such as foreign drug names, typographical errors, unspecified names (e.g., BLINDED PLACEBO), herbs (e.g., ALOEELITE), domestic drugs (e.g., PANHEPRIN), new drugs (e.g., HIZENTRA, approved by the FDA in 2010), and non-drugs (e.g., RADIATION THERAPY). A small portion of them (25) that failed to be mapped are due to MedEx. The evaluation results revealed several issues related to drug name normalization. First, the public release of MedEx (version 2.0) is in an executable format, which prevents the use of the latest version of RxNorm for drug name normalization. For example, the drug name “SAPHRIS” did not have a match using MedEx because it is included in RxNorm in the 2012 version but not in the 2008 version (used in MedEx). Second, RxNorm does not contain foreign brand names since it is intended to cover drugs prescribed in the United States. Third, we found that some of the records in AERS contain unspecified names. An example is the name “BLINDED PLACEBO,” which is used as a drug name and makes the normalization infeasible. We would suggest that incorporating thorough normalization at the point of data entry is desired, so as to improve the data quality for data mining.

**Table 6:** Reasons for non-normalization of drug names by MedEx

**Reasons**	**No.**
**MedEx reasons**	**25**
**AERS entry reasons**	**75**
Foreign brand names	41
Typo	19
Unspecified names	8
Herbs	4
Uncovered domestic drug	1
New drug	1
Non-drug	1
Total	100

Manual evaluation shows the greedy algorithm used to find mapping between RxNorm and NDF-RT is 100% valid.

### Case studies for AERS-DM

**Figure 6:**
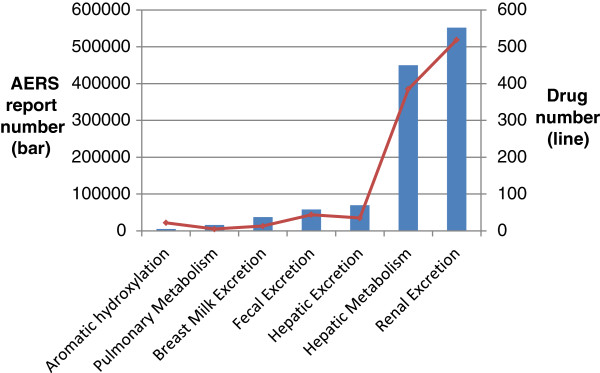
A profile of pharmacokinetics classes in AERS.

**Figure 7:**
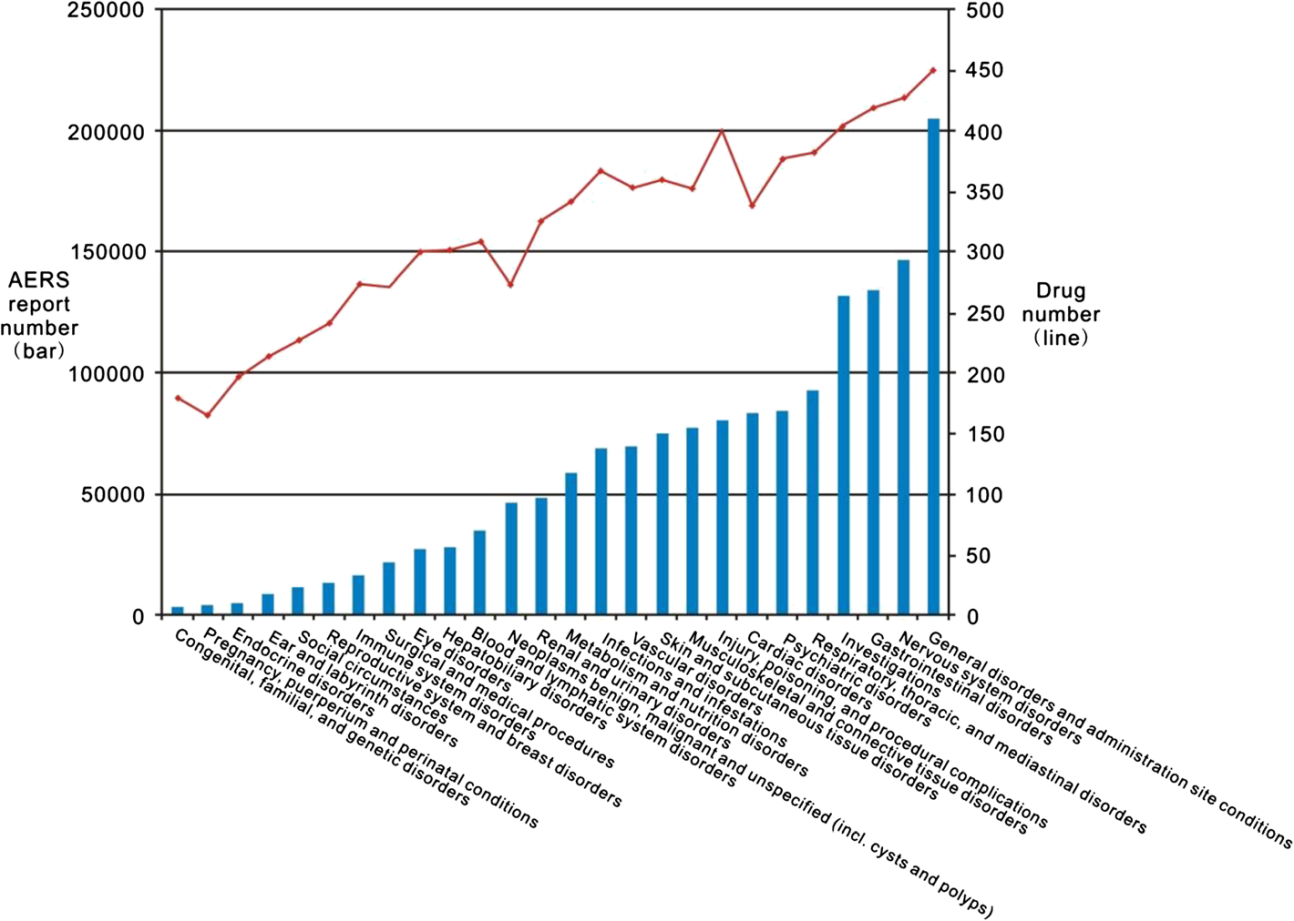
A profile of MedDRA SOC for pharmacokinetics classes in AERS.

As described in the section above, the study produced an AERS-DM containing normalized and aggregated AERS reporting data. To demonstrate the usefulness of the AERS-DM, we used the AERS-DM to analyze the NDF-RT drug class “Pharmacokinetics” and their corresponding ADE categories represented by the MedDRA SOCs. Specifically, we joined the two tables in the AERS-DM and retrieved the AERS reports under seven existing pharmacokinetics classes. We then analyzed the data, including their corresponding MedDRA SOC-based ADE categories.Figure 6 shows a profile of seven pharmacokinetics classes in the AERS-DM. The bars represent the total number of AERS reports for each individual pharmacokinetics class and the line represents the total drug numbers under each pharmacokinetics class. This figure illustrates that most drugs with pharmacokinetics class information reported in the AERS are relevant to “Renal Excretion” and “Hepatic Metabolism.” We also found that the number of AERS reports is disproportional to the number of drugs for some pharmacokinetics classes. For example, the class “Hepatic excretion” contains fewer drugs than the class “Fecal excretion” but has more AERS reports. The result indicates that the drugs in the “Hepatic excretion” class may be associated with more AERS reports than the “Fecal excretion” class, and interesting etiology knowledge may be found through further mining with disproportionality metrics and other data sources, including EHRs.Figure 7 shows a profile of 26 MedDRA SOC-based ADE categories that corresponds to the seven pharmacokinetics classes in the AERS. The bars represent the total number of AERS reports for each individual SOC and the line represents the total number of drugs associated with each SOC category. The figure illustrates that the top five most frequent ADE categories are “General disorders and administration site conditions,” “Nervous system disorders,” “Gastrointestinal disorders,” “Investigations,” and “Respiratory, thoracic, and mediastinal disorders.” Similarly, we found that the number of AERS reports is disproportional to the number of drugs for some MedDRA SOC categories. The result may reveal some important areas of ADE surveillance for post-marketing drugs if combined with prescription information as the denominator.

## Discussion

In addition to the significance described in the Introduction section above, the present study was also partially motivated by our previous work on building a standardized knowledge base of ADEs known as ADEpedia, in which we intended to integrate and normalize known ADE knowledge from disparate ADE datasets (e.g., the FDA structured product labels, and the UMLS) [35,36]. We consider that the FDA AERS reporting database is another important data source for ADE knowledge discovery and the normalization of both the drug and ADE names is the first important task we need to tackle with.

### Drug and ADE aggregation

NDF-RT is a major national source of drug classification information, providing multi-axial classifications such as physiological effect, mechanism of action, etc. It has proved to be capable of representing medications in clinical settings. For example, Rosenbloom et al. [37] investigated the coverage of the Physiologic Effects hierarchy in NDF-RT and found this category to be sufficient for classifying medications. Zhu et al. [38] used the FDA Established Pharmacologic Class in NDF-RT to profile Structured Product Labelling for clinical applications.

On the other hand, the applicability of NDF-RT and RxNorm for clinical drug classification was explored, and the imperfect mappings between RxNorm and NDF-RT and incomplete drug classification were evidenced by several studies [22,24,25]. Palchuk et al. [25] used the NDF-RT’s drug class tree to organize RxNorm into a hierarchy and evaluated this mapping using data from EHRs. Pathak et al. [22] investigated the applicability of RxNorm and NDF-RT for representation and classification of medication data from EHRs using the NLM’s NDF-RT web services API for NDF-RT drug class assignment. Both of the above studies were limited to the “Drug Products by VA Class” hierarchy under “Pharmaceutical Preparations,” with no consideration of the multi-axial hierarchies. In addition, issues in mapping and classifying drugs from RxNorm using the NDF-RT’s multi-axial classification were investigated by Pathak and Chute [24]. In the study, they identified the issues in NDF-RT, including the lack of coverage of drug classes (chemical structure, mechanism of action, physiologic effect, therapeutic intent, and pharmacokinetics) for clinical drugs, and suggested that the resolution would rely on the targeted improvement of NDF-RT. Thus, the existing studies on the classification of RxNorm using NDF-RT are either about multi-axial classification, based on the mapping between the two ontologies, or limited to VA class based on EHR data.

In the context of standardizing the AERS data in this study, we developed a systematic algorithm in which the rich semantic connections within RxNorm were fully utilized to build the mappings with the related concepts in NDF-RT. The mappings were then used to aggregate the AERS data under multi-axial classifications in NDF-RT. Different from those related studies, the present study focuses on real-world data in the AERS and presents a normalized AERS-DM data set together with all corresponding drug classification information provided by NDF-RT for large-scale data mining purposes. The evaluation results show that the greedy algorithm for maximum mapping to corresponding NDF-RT concepts from RxNorm codes was valid. We believe that the mapping method developed in our study could be useful in other similar context.

We found that the MedDRA PT terms coding ADEs in the AERS changed over time. The used AERS data ranged from 2004 to 2011, and during that time period the MedDRA versions had been updated twice annually [39]. Some ADE codes in the AERS from the older MedDRA edition had become obsolete and may hinder the identification of Drug-ADE pairs. In the future, we will consider whether making the PT terms consistent over the years before conducting ADE detection (i.e., building a version control mechanism) may be useful for improving data quality.

In addition, other studies have identified a number of issues related to the use of MedDRA. For example, the hierarchy problems and semantic reasoning incapability of MedDRA mitigate its usefulness for querying and analyzing AERS data. SNOMED-CT, as the largest clinical terminology, can complement these disadvantages, with as many levels of hierarchy as are considered appropriate, and the semantic consistency in relationships [40]. In addition, using SNOMED-CT for ADE coding can also achieve the integration of ADE data in the AERS with other health data sources, including EHRs. A few studies have demonstrated the mappings between MedDRA and SNOMED-CT. For example, Bodenreider [41] proposed the mappings by leveraging the structure of SNOMED-CT for aggregation purposes. Mougin et al. [42] proposed to improve the mapping through an automatic lexical-based approach. A recent study [43] compared three methods using the Ontology of Adverse Events (OAE), MedDRA, and SNOMED-CT in classifying the ADE terms associated with two vaccines. Among the three methods,, the OAE method provided better classification results. This initiative is inspiring in the field of ADE detection for vaccines. However, given that it is a newly emerging ontology with only 2723 terms, the coverage of the OAE is very limited. For the AERS data normalization and aggregation, we consider that the widely used SNOMED-CT would be a better candidate as an ADE terminology, and this will be one of our future works.

### Case studies

We demonstrated the usefulness of the AERS-DM produced by this study by analyzing the data set using the pharmacokinetics class. There are other class dimensions available for analysis, including physiologic effect, mechanism of action, and VA class, all of which come from the knowledge structure asserted in the NDF-RT. We believe that the knowledge asserted in the standard ontologies will enrich the AERS-DM and enable the meaningful use of AERS data for ADE signal detection and data mining.

### Implication of study

Based on AERS-DM, more efficient data mining and ADE detection in individual drugs would be achieved and facilitated. The reasons are three-fold as follows. First, having enriched features of drugs and enlarged cohort information, AERS-DM could provide potential explanations for individual differences in ADEs. Second, with the capability of large-scale ADE mining, comparative analysis of different drug classes could be explored, thus accumulating ADE evidence in the field of individual drugs. Third, presenting more meaningful organization of drug and ADE data with standard terminologies, AERS-DM could be a platform for deeper mining by further connecting with clinical notes, scientific literature, gene expression, proteomics and pharmacogenomics data, and various other ontologies. We believe that AERS-DM could be used to explore the complex network among drugs and ADEs, and such research would bear far-reaching significance in terms of the study paradigm of ADEs.

### Limitations

We used only two drug ontologies (RxNorm and NDF-RT) and one ADE terminology (MedDRA) to normalize and aggregate AERS data. We believe additional investigations of other standard terminologies, such as SNOMED-CT would be beneficial in exploring the potential of standardized AERS reporting data in data mining.

## Conclusion

In this study, we leveraged three biomedical ontologies―RxNorm, NDF-RT, and MedDRA―for normalizing and aggregating the AERS data and produced a standardized ADE dataset referred to as AERS-DM. With the normalized codes and aggregated features, the AERS-DM would be useful for the research community in the data mining field. We will continue to refine and optimize the AERS-DM and update it periodically in the future. In addition, we will investigate the integration of the AERS-DM data set with other health data sources, such as EHR data, literature databases (e.g., Semantic Medline [44]) and other ontologies (e.g., Drug Ontology [45]), for the purpose of promoting ADE detection in individual drugs. Finally, we will leverage SNOMED-CT for standardizing the AERS data.

### Availability and requirements

**Dataset name:** AERS-DM

**Dataset home page:**http://informatics.mayo.edu/adepedia/index.php/Download

**Operating system(s):** Platform independent

**Other requirements:** None

**License:** GPL

**Any restrictions to use by non-academics:** None

**RxNorm Files:**http://www.nlm.nih.gov/research/umls/rxnorm/docs/rxnormfiles.html

**RxNorm concept relationships:**http://www.nlm.nih.gov/research/umls/rxnorm/docs/2013/appendix1.html

## Abbreviations

AERS: The Adverse Event Reporting System; NDF-RT: National Drug File-Reference Terminology; MedDRA: Medical Dictionary for Regulatory Activities; PT: Preferred Term; SOC: System organ class; HGLT: High-Level Group Term; HLT: High-level term; LLT: Lowest-level term; ADEs: Adverse drug events; AERS-DM: AERS data mining set; EHRs: Electronic health records; PRR: Proportional reporting ratio; ROR: Reporting odds ratio; IC: Information component; EBGM: Empirical Bayes geometric mean; RRF: Rich Release Format; NLM: National Library of Medicine; RxCUI: RxNorm concept unique identifier; UMLS: Unified Medical Language System.

## Competing interests

The authors declare that they have no competing interest.

## Authors’ contributions

All co-authors are justifiably credited with authorship, according to the authorship criteria. Final approval is given by each co-author. In detail: LW –design, development, analysis of data, interpretation of results, and drafting of the manuscript; GJ – conception, interpretation of results, and critical revision of the manuscript; DL – analysis of data; HL – conception, design, development, interpretation of results, and critical revision of manuscript.
